# Navigation of an Autonomous Spraying Robot for Orchard Operations Using LiDAR for Tree Trunk Detection

**DOI:** 10.3390/s23104808

**Published:** 2023-05-16

**Authors:** Ailian Jiang, Tofael Ahamed

**Affiliations:** 1 Graduate School of Science and Technology, University of Tsukuba, 1-1-1 Tennodai, Tsukuba 305-8577, Japan; s2236016@u.tsukuba.ac.jp; 2 Institute of Life and Environmental Sciences, University of Tsukuba, 1-1-1 Tennodai, Tsukuba 305-8577, Japan

**Keywords:** LiDAR, tree trunk detection, orchard

## Abstract

Traditional Japanese orchards control the growth height of fruit trees for the convenience of farmers, which is unfavorable to the operation of medium- and large-sized machinery. A compact, safe, and stable spraying system could offer a solution for orchard automation. Due to the complex orchard environment, the dense tree canopy not only obstructs the GNSS signal but also has effects due to low light, which may impact the recognition of objects by ordinary RGB cameras. To overcome these disadvantages, this study selected LiDAR as a single sensor to achieve a prototype robot navigation system. In this study, density-based spatial clustering of applications with noise (DBSCAN) and K-means and random sample consensus (RANSAC) machine learning algorithms were used to plan the robot navigation path in a facilitated artificial-tree-based orchard system. Pure pursuit tracking and an incremental proportional–integral–derivative (PID) strategy were used to calculate the vehicle steering angle. In field tests on a concrete road, grass field, and a facilitated artificial-tree-based orchard, as indicated by the test data results for several formations of left turns and right turns separately, the position root mean square error (RMSE) of this vehicle was as follows: on the concrete road, the right turn was 12.0 cm and the left turn was 11.6 cm, on grass, the right turn was 12.6 cm and the left turn was 15.5 cm, and in the facilitated artificial-tree-based orchard, the right turn was 13.8 cm and the left turn was 11.4 cm. The vehicle was able to calculate the path in real time based on the position of the objects, operate safely, and complete the task of pesticide spraying.

## 1. Introduction

Driven by rapid socioeconomic development and urbanization, young populations are leaving rural areas to work in other industries in cities [1]. In Japan, more than 60% of the farmer population is over the age of 65. Japan’s rapidly aging agricultural society will not only reduce Japan’s food self-sufficiency but also lead to a downturn in domestic agricultural business, which may reduce revenues [2]. To solve the problems of an aging population and labor shortages, many machines have been developed to assist in agricultural production. With the development of this machinery, agriculture has become less dependent on human labor. Especially in the areas of extensive farming and fixed planning areas, advanced automation has the opportunity to bring time efficiency and high economic benefits because of timeliness and efficiency in operation. Crops that require pesticides and herbicides can affect human health, and machinery is a necessary alternative to ensure flexibility and safety [3]. Pesticide spraying is essential; however, there is a high risk of chemical contamination due to overapplication of conventional speed sprayers. From flowering to fruiting, pesticide spraying needs to be repeated several times to protect from pest invasion. In some cases, farmers also spray pesticides manually, and pesticides can enter farmers’ bodies through their respiratory systems, affecting their health. Furthermore, most orchards in Japan are conventional, and farmers control the height of tree growth according to their operations, causing limited operating space in the orchard, and the branches not only scratch the machinery but also block the driver’s view, thus making it unfavorable to operate medium- and large-sized machinery in orchards due to hampered navigation. Therefore, compact and unmanned agricultural machinery has the potential to overcome the problem of maneuvering with an appropriate autonomous guidance system.

In an autonomous agricultural guidance system, navigating with accurate positional information through sensors and surrounding environmental information is required for vehicle movement in the designed path. In agricultural automation and robotics applications, vehicle navigation is important in outdoor environments, which are complex and uncertain compared to indoor conditions [4]. An orchard navigation system includes three parts: environment detection, path planning, and navigation control [5]. Currently, the main sensors used for environmental sensing are the global navigation satellite system (GNSS), machine vision, light detection and ranging (LiDAR), and multi-sensor fusion. The GNSS offers the highest accuracy in open field navigation and has brought significant success using the Real Time Kinematic Global Navigation Satellite System (RTK-GNSS) with higher accuracy [6]. However, the performance of GNSS-based navigation depends highly on GNSS signal quality; orchard navigation is the most complex, and interruptions occur in RTK-GNSS signals because high and dense canopies are frequently encountered [7]. Because Japanese orchards use nets and are compact with dense branches, GNSS signals can be affected, and many farmland orchards do not have base stations set up to use GNSS directly.

Conventional machine turning is also difficult to achieve with GNSS-based oriented sensors. A machine-vision-based navigation system relies on a camera to acquire images in real time, obtains the robot’s position and attitude through feature clustering, threshold segmentation or path line extraction, and finally generates control signals for robot movement and steering. With the advantages of high flexibility, rich and complete information acquisition, and low influence from complex environments, machine vision is widely used in the field of agricultural navigation [8,9]. This method requires a clear contrast between the fruit trees and the background features on the ground, but high and dense weeds commonly grow at the bottom of the fruit trees, making the boundary between the trees and the ground shift or even undetectable. Environmental light has a large impact on machine vision; in the dark or at night, the accuracy of machine vision is low, and even in the daytime, the shadows of surrounding plants (such as weeds) also affect the accuracy [10]. Due to the complex and variable agricultural environment, the use of only one sensor technology is increasingly unable to meet the accuracy and stability performance of navigation systems. In this case, multi-sensor fusion technology has been introduced to combine several sensors into a system to fuse data and obtain more accurate navigation and positioning information [11]. To obtain the absolute position and absolute heading of a robot, the inertial measurement unit (IMU) is often used in combination with the RTK-GPS to improve system robustness [12,13]. However, the RTK-GNSS and IMU systems together also increase algorithmic complexity and sensor costs, even though it is difficult to reach reliability in orchard navigation systems. In contrast, LiDAR is less affected by external environmental conditions, more resistant to interference, can directly obtain obstacle depth information, and has the characteristics of fast processing speed to satisfy real-time detection. LiDAR cloud points and pattern matching with machine learning algorithms have significant potential to narrow down the complexity of the navigation system.

LiDAR is used to scan the surrounding environment in real time and returns accurate distance information using the time-of-flight principle. Laser navigation has advantages such as high ranging accuracy, good resolution, and strong anti-interference ability [14]. LiDAR can overcome the limitations of low light and the interruption of RTK-GNSS signals. There are several methods used to process LiDAR data. The density-based clustering algorithm (DBSCAN) is capable of detecting arbitrary shapes of clusters in spaces of any dimension, and this method is very suitable for LiDAR data segmentation [15]. To set the parameter searching radius and to make it more consistent with different point cloud environments, automatic searching radius *ε* estimation was proposed based on the average of nearest neighbors’ maximum distance [16]. In line detection, 2D point clouds returned by LiDAR use several algorithms such as, random sample consensus (RANSAC) [17,18], Hough transform [19,20], the least squares method [21], and a line segment detector (LSD) [22]. Simultaneous localization and mapping (SLAM) technology developed the GNSS-independent VineSlam localization mapping method and the vineyard-specific path planner “AgRobPP” was reported. This method provided good results in mapping and path planning and could control the robot to perform a variety of tasks [23]. However, 3D LiDAR point cloud data required extensive processing of computational costs and computational requirements [24]. Therefore, Saike et al. processed 3D LiDAR data in 2D to achieve autonomous navigation operations in a greenhouse [25].

Therefore, the purpose of this study was to develop a compact robot for spraying in orchards using LiDAR for tree trunk detection. To develop a single sensor-based navigation system, fusion of several machine learning algorithms is proposed to achieve a navigation system that is not affected by lighting conditions. Trunk detection can be performed using LiDAR to achieve a navigation system that is not affected by lighting conditions. Considering that the DBSCAN algorithm has the potential to exclude noise points through the density clustering of points, this study first used the DBSCAN algorithm to process the 2D point cloud returned by LiDAR to obtain the object’s position. Since the trees in the orchard are planted in columns, the trees can be used as a reference for path planning based on the positions of the left and right sides. Therefore, in the second step, the K-means algorithm is described to divide the objects into two groups on the left and right. Some trees in the orchard may be surrounded by shelves, and to reduce the influence of these factors on the path planning results, in the third stage, this study used the RANSAC algorithm to exclude the influence of noise points on the boundary line calculation as much as possible.

In the Materials and Methods section, the hardware system architecture used in the study and the algorithms used for LiDAR navigation is introduced, as well as the software environment. The Results section discusses the feasibility of the method proposed in this study based on several experiments on different sites. In the Discussion section, the results of the study are analyzed and future modifications are proposed to overcome deficiencies of the proposed method and prototype. The final section concludes the paper and presents perspectives on the proposed developments and further recommendations.

## 2. Materials and Methods

In this research, the methodology was divided into two sections: the small and compact prototype development and the navigation system using LiDAR based on several machine algorithms to process cloud points for pattern matching and path planning in orchards.

### 2.1. Experimental Prototype Vehicles and the Installation of Sensors

#### 2.1.1. Vehicle

**Figure 1:**
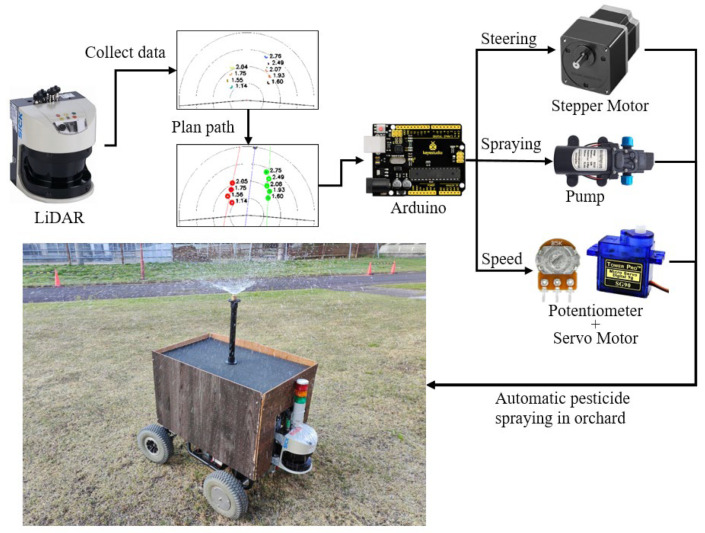
Two-dimensional LiDAR-based navigation system design for facilitated orchards using a converted electrical vehicle as a spraying robot.

An EJ20^®^ electric cart (CANYCOM, Fukuoka, Japan) was the experimental vehicle, which was configured as the prototype vehicle and modified for a Japanese compact orchard to provide the opportunity for easy maneuvering. This product was originally used to assist workers in carrying goods, which requires manual start and control of its direction and has no automatic moving function; however, this vehicle has a complete motor drive control system. Therefore, the electric car was modified for control using a microcontroller-based command system (Figure 1). This vehicle was controlled by steering the rear wheels; therefore, to realize automatic steering, a stepper motor (pk296a1-sg36^®^, Oriental Motor Co., Ltd., Tokyo, Japan) was installed at its steering rear axle and driven by a stepper motor driver (TB6600, ViaGasaFamido, Shenzhen, China). This reduction stepping motor has a maximum torque of 12 N·m, and even in the soil, the torque can be increased by reducing the rotation speed to provide enough force to control the vehicle steering. The original vehicle was controlled by a rotary potentiometer (B5K, Keyestudio, Shenzhen, China), which was used to control the start of the vehicle as well as the speed by dividing the voltage. This study connected the rotary potentiometer and the servo motor (SG90, Keyestudio, Shenzhen, China) to achieve accurate speed control by using signals. The orchard system was facilitated using artificial trees for forward movements and turning inside rows.

**Figure 2:**
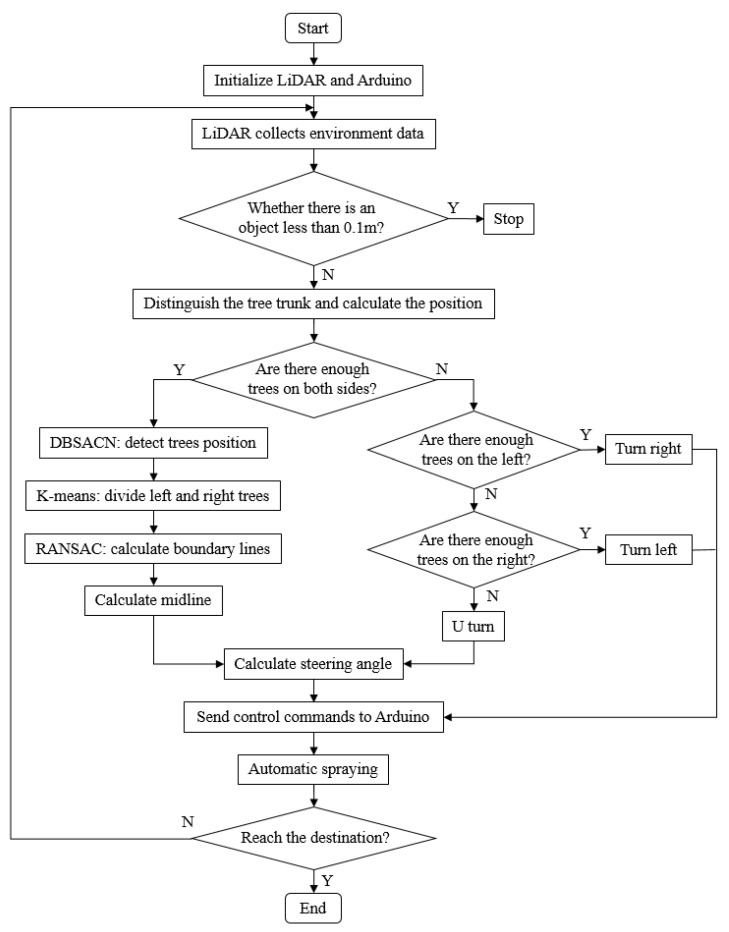
Operational flow of the sensors and 2D LiDAR to enable the spraying system to work.

The system was developed using a microcontroller to send different commands to Arduino (UNO R3, Keyestudio, Shenzhen, China) and to control the motor, potentiometer, and spraying device to complete the task of automatic pesticide spraying in orchards (Figure 2).

#### 2.1.2. Pesticide Spraying System

A pesticide spraying system was developed using a pesticide tank that was located on the vehicle, and the pump was installed inside the tank. The pump was controlled using an Arduino control relay to turn on/off the pump. The nozzle was installed on the top of the vehicle. This vehicle was equipped with a signal tower controlled by two channel relays: a green light when the vehicle was safe to operate and a red light when the robot malfunctioned or stopped.

#### 2.1.3. LiDAR

**Figure 3:**
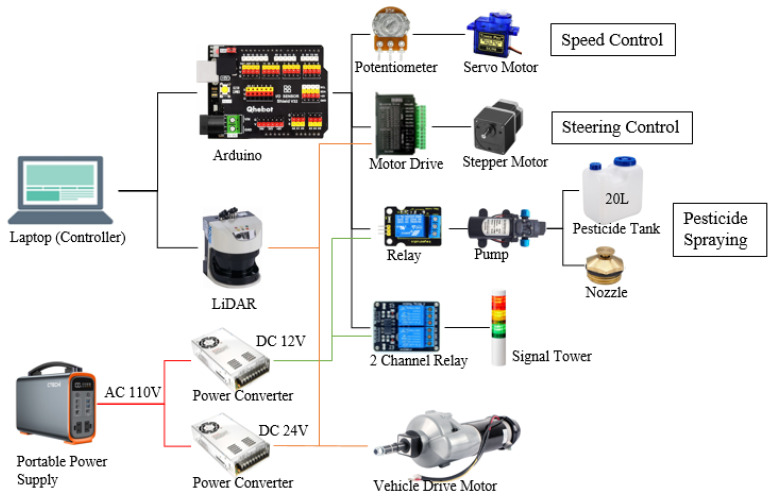
Sensors and connection for the robotic system.

A LiDAR system (SICK LMS511-20100 PRO^®^, Waldkirch, Germany) was chosen as the sensor in this study. The LiDAR system was installed at the front of the vehicle to detect the road (Figure 3). A 24 V power supply unit was used as the power converter (200305_JPN, DROK, Guangzhou, China). The LiDAR was operated with an angular scanning range of 190°, and the resolution was 0.25°. The working distance of the LiDAR system was designed within a range of 80 m. Calibration was performed to confirm the highest accuracy of detection. After receiving the command, the LMS511 emitted infrared rays from right to left in each 0.25° interval starting from −5°, and the distance was calculated based on the time difference of infrared ray return, which returned 761 points of distance in one scan. The LiDAR system can only obtain the distance value of each point; therefore, it is necessary to cluster the points by using an algorithm to find the position of the objects and plan the path.

### 2.2. Path Planning Algorithm

**Figure 4:**
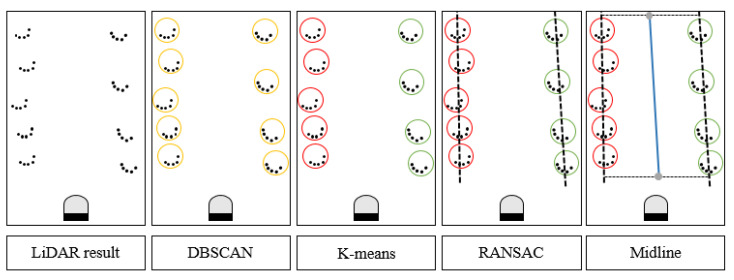
Path planning algorithm from cloud point clusters received from the 2D LiDAR system.

In this study, three algorithms were used to process the LiDAR-returned cloud points sequentially. First, the returned points were clustered by the density-based spatial clustering of applications with noise (DBSCAN) to find the tree trunk position. K-means was used to discriminate between the left and right sides of the tree. Two straight lines were calculated as the boundary lines using random sample consensus (RANSAC). Finally, the midline was calculated as the planning path for navigation (Figure 4).

#### 2.2.1. Density-Based Spatial Clustering of Applications with Noise (DBSCAN)

DBSCAN is a density-based clustering algorithm consisting of two parameters: the radius ε and the minimum number of points minPoints. Through DBSCAN clustering, the points were classified as core points, border points and noise points. The neighborhood of p was the set of neighbors within distance ε, denoted by Nε(p). The number of points in p’s ε-neighborhood was denoted by |Nε(p)|.

- Core points: If |Nε(p)| ≥ minPoints, then p is a core point, and all the points in this set, together with p, belong to the same cluster.
- Border points: They are directly reachable from the core point; however, |Nε(p)| < minPoints belongs to the same cluster with the core point.
- Noise points: A point that is not included in any cluster.

**Figure 5:**
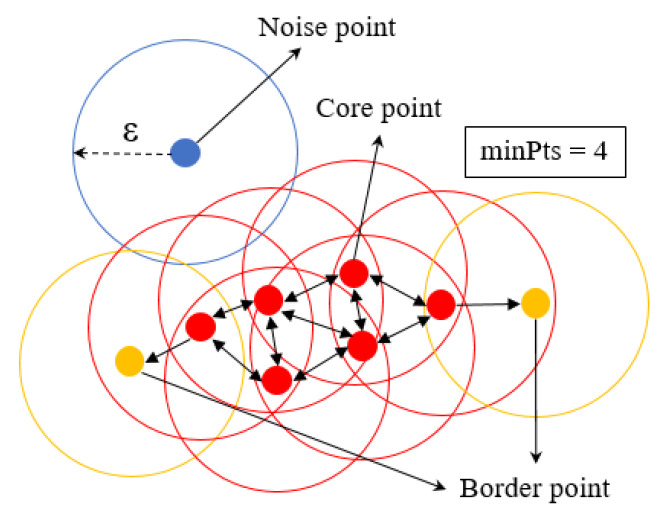
DBSCAN algorithm for core points of the object from the cloud points of the 2D LiDAR system.

The DBSCAN algorithm returned all clusters by checking the neighborhood of each point in the dataset in an arbitrary order. If p was a core point, a new cluster C containing p was created. Then, the new cluster was expanded by iteratively adding non-clustered points that could be directly density-reachable from at least one point in C. The expansion of C finished when all density-reachable points from p were added to C. The DBSCAN algorithm ended with every point in the dataset being assigned to a cluster or marked as a noise point (Figure 5) [26].

#### 2.2.2. K-Means Clustering

**Figure 6:**
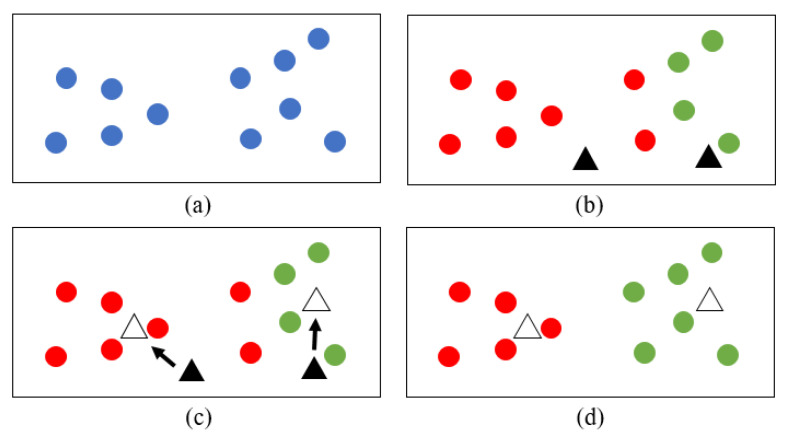
Diagram of the K-means algorithm for clustering two sets of cloud points on the left and right sides of the midline of the 2D LiDAR system. (**a**) Cloud points that need to be clustered. (**b**) First clustering is based on random virtual cluster centers. (**c**) Calculate the new virtual cluster center based on the previous clustering result. (**d**) By multiple iterations, the clustering result of the closeness minimum value is the final result.

The K-means clustering algorithm is an unsupervised method designed to split a given unlabeled data distribution into a fixed number K of clusters in which each group shares common characteristics [27]. This algorithm was used to minimize intraclusteral variance, which can be formulated as expression (1) based on optimization [28]. After classification, each class existed as a virtual cluster center *u_i_*. Therefore, the closeness (*E*), which represents the closeness of the sample around each clustering center, can be expressed as:

(1) $$E=\overset{k}{\underset{i=1}{\sum }}\underset{S\in C_{i}}{\sum }\|X_{n}-u_{i}\|$$

where given a sample set *S*, the set can be expressed as *S* = {*X_1_, X_2_, …, X_n_*}, where *n* is the total number of samples. *u_i_* is the sample mean of class *i*. In this research, K-means was used to divide the trunk into left and right clusters; thus, the *i* value was 2 (Figure 6). *Ci* is the set of class *i*. The smaller E is, the higher the similarity of the clustered samples. The determined number of clusters, clustering centers, and the traversal order of samples affected the accuracy of the K-means clustering algorithm.

#### 2.2.3. RANSAC

**Figure 7:**
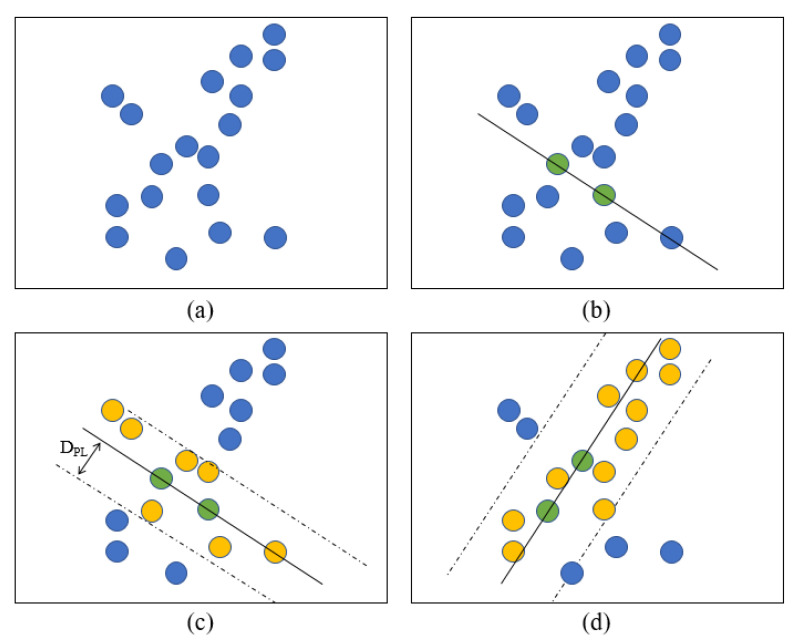
Diagram of the RANSAC algorithm for determining the boundary line of cloud points received from the 2D LiDAR system. (**a**) Cloud points that need to be clustered. (**b**) Randomly select two points to make a line L. (**c**) The distance D from data points to line L was calculated, if D < D_PL_, the point was in line (yellow point); otherwise, it was classified as out of line (blue point). (**d**) After several iterations until ProT_current was greater than the set ProT, the calculation was completed.

RANSAC is a data clustering algorithm, which is a density-based clustering nonparametric algorithm. This algorithm simply iterates two steps: generating a hypothesis by random samples and verifying the hypothesis with the remaining data to estimate the mathematical model from a set of data containing outliers [29]. RANSAC needs to set three parameters before operation: the threshold value, which is the distance from the point to the line (D_PL_), the inline point proportion threshold (ProT), and the maximum operation times Max_Number. For the calculation, the first two points were randomly selected to match line L, and then, the formula of L was obtained. The distance D from all data points to line L was calculated, and if D < D_PL_, the point was in line; otherwise, it was classified as out of line, and the in-line proportion of ProT_current was calculated after assigning all the points. The proportion was compared with the saved best proportion, and the larger proportion was recorded. When the proportion was greater than the set ProT or reached the set maximum operation time, the calculation of the RANSAC algorithm was completed (Figure 7).

### 2.3. Vehicle Guidance

**Figure 8:**
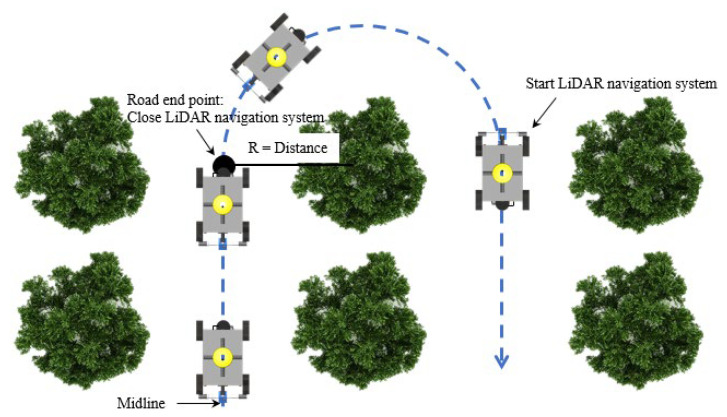
U-turn of the field experiment for the navigation of the experimental robots (the road end point is the center point of the last two trees). The steering path was planned according to the distance from the tree trunk.

The vehicle was guided at the forward speed until it reached the end of the tree rows, and the number of tree trunks was not enough to use the above method for trajectory planning. In this study, when the vehicle was operated at the road end point (the midpoint of the last two trees), the vehicle steering angle was set based on the distance to the trunk from the LiDAR system (Figure 8). Vehicle steering was started, and the LiDAR system continued to scan the surrounding environment information. DBSCAN and K-means were used to continue to distinguish the location of the surrounding trunks. Until the vehicle was turned to the next road in which the left and right sides obtained enough points returned from the tree trunk, the steering ended and continued to operate according to the path planning algorithm.

### 2.4. Control System

#### 2.4.1. Trajectory Tracking Control

The path tracking of the robot was divided into three types according to the model used: kinematic-model-based path tracking, dynamic-model-based path tracking, and path tracking that does not require a model. The mathematical model based on dynamics requires a force analysis of the tires and many considerations of the farmland soil. Most of the parameters in the dynamic principle are difficult to determine, leading to a mathematical model that is challenging. The kinematic-model-based path tracking method determines the turning direction and angle of the robot based on the robot’s current position and heading parameters. Therefore, the robot could travel along the established route. The kinematic or dynamic-model-based path tracking method has a negative impact on the robot’s path tracking performance due to inaccurate modeling or drastic changes in model parameters. The pure pursuit tracking method was used in this study, which did not require a model. The pure pursuit tracking model is a model that describes the path tracking of a robot with a fixed forward visual distance using geometric equations. The model is simple, intuitive, and easy to implement. The core purpose of the model is to determine a suitable forward-looking distance.

**Figure 9:**
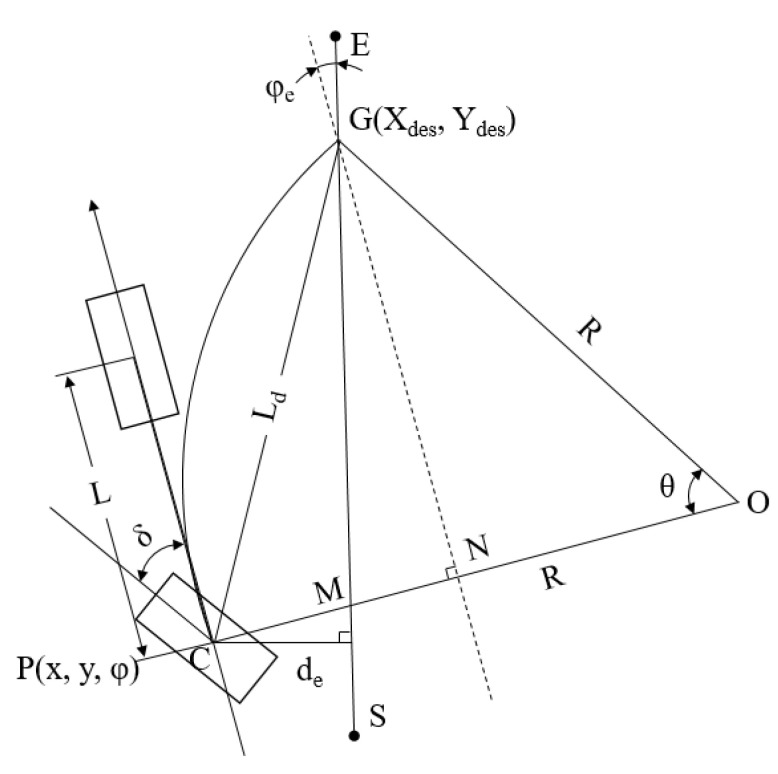
Pure pursuit tracking model of the experimental robot.

The experimental robot had a four-wheel drive and rear-wheel steering system. A simplified bicycle model was used in the pure pursuit tracking model in the concept of a geometry schematic. The robot rear axis center point *C* is shown as the current position of the robot, *O* is the instantaneous circle center when the robot was steered, and *R* is the instantaneous steering radius (Figure 9). The target point *G* on the planning path was selected as the current target position, *L_d_* was the forward-looking distance from the center point *C* to the target point *G*, the arc length of *CG* was the path that the robot needed to move to the target position, and *δ* was marked as the theoretical steering angle required by the robot. In the next cycle, the robot targets a new driving arc based on the current position and the target position and calculates a new theoretical steering angle *δ*. The current position can be infinitely close to the target position.

In the pure pursuit tracking system, the robot’s current position was represented by *P(x, y, φ)*, *d_e_* was the trajectory tracking horizontal error, *φ_e_* was the heading error, and *θ* was the change angle while the robot was steered to the target point *G*. Based on the geometric relationship, it can be determined that:

(2) $$L_{CN}=R-Rcos\theta$$

(3) $$L_{NG}=Rsin\theta$$

(4) $$L_{CN}^{2}+L_{GN}^{2}=L_{d}^{2}$$

(5) $$L_{CN}=d_{e}cos\varphi_{e}+\sqrt{L_{d}^{2}-d_{e}^{2}}sin\varphi_{e}$$

Equations (2)–(4) are simplified to obtain:

(6) $$R=\frac{L_{d}^{2}}{2L_{CN}}$$

The relationship between the instantaneous steering radius, steering angle, and wheelbase is:

(7) $$\tan\delta =\frac{L}{R}$$

Equations (5)–(7) are used to obtain:

(8) $$\delta =\arctan\frac{2L\left(d_{e}cos\varphi_{e}+\sqrt{L_{d}^{2}-d_{e}^{2}}sin\varphi_{e}\right)}{L_{d}^{2}}$$

Equation (8) shows that after determining the suitable forward-looking distance *L_d_*, the robot’s front wheel angle δ was directly determined by the horizontal error de and heading error *φ_e_*. These two values were obtained from the distance and angle to the LiDAR planning path.

#### 2.4.2. Navigation Decision

PID control systems are widely used in industrial process control due to their simple principle, high robustness, and wide practicality. The PID control system uses proportional (P), integral (I), and derivative (D) controllers to obtain a desired response [30]. The output of the controller is the sum of a proportional term, an integrating term, and a differentiating term, with an adjustable gain for each term [31]. The PID output *u(t)* can be expressed as in expression (9):

(9) $$u\left(t\right)=K_{p}e\left(t\right)+K_{i}\int_{0}^{t}e\left(\tau \right)d\tau +K_{d}\frac{d}{dt}e\left(t\right)$$

*K_p_*, *K_i_*, and *K_d_* are all nonnegative and denote the coefficients for the proportional, integral, and derivative terms, respectively. *e* is the error between the set value and the feedback value. In PID control, the proportional part only considers the current error of the robot in the trajectory path; if the system has deviations, the proportional controller can react quickly to reduce the error. The integral controller adds the accumulated error to the original system to cancel the deviation that occurs when the system is stable. The differential controller is based on the trend of the system and adjusts ahead according to the trend of the deviation signal.

However, the integration part occupies considerable computational capacity due to accumulating all the errors, so incremental PID control uses increments instead of the integration part; the incremental PID can be expressed as expression (10):

(10) $$u\left(t\right)=K_{p}\Delta e\left(t\right)+K_{i}e\left(t\right)+K_{d}\left[\Delta e\left(t\right)-\Delta e\left(t-1\right)\right]\;where\;\Delta e\left(t\right)\;=e\left(t\right)-e\left(t-1\right)$$

Compared to PID control systems, incremental PID control reduces the number of calculations because the output results are only related to the last three errors, which does not seriously affect the operation even if there are serious errors in the system.

#### 2.4.3. Program Platform and GUI

**Figure 10:**
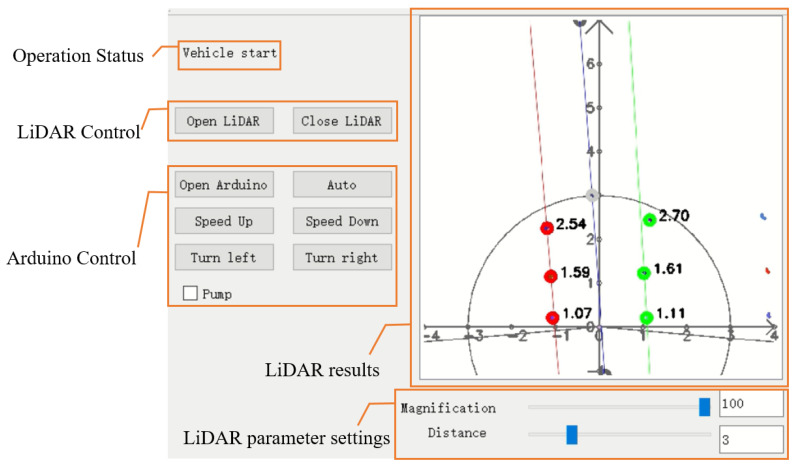
Graphical user interface to control the LiDAR system and the microcontroller and visualization of cluster datasets from cloud data points.

This study used C++ programming on Visual Studio 2017 to achieve communication with LiDAR and Arduino. To facilitate control and make the results more intuitive, Qt^®^ was used to build the graphical user interface (GUI). The GUI included control buttons for LiDAR and Arduino, showing the program operation status and LiDAR planning path result and the setting of LiDAR parameters. The magnification and scanning range of the LiDAR result image could be modified by sliding. The black circle in the result image represents the scanning range, and only the data inside the circle are used for path planning (Figure 10).

## 3. Results

### 3.1. Planning Path Calibration on a Concrete Road

**Figure 11:**
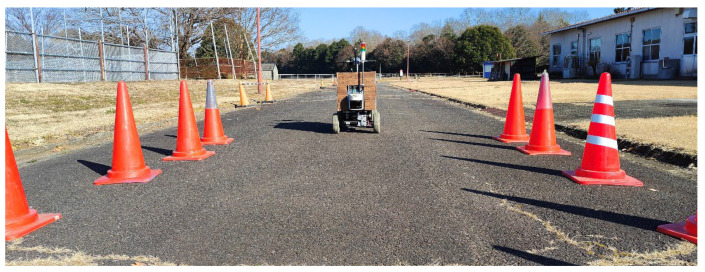
Calibration of the LiDAR sensor and the robot on the concrete road (cones were used instead of trees).

This calibration was conducted on a concrete road using cones instead of tree trunks. The positions of the cones simulated the planting of trees in an orchard, approximately in a line vertically, and the trunks were not in the same line horizontally (Figure 11).

**Figure 12:**
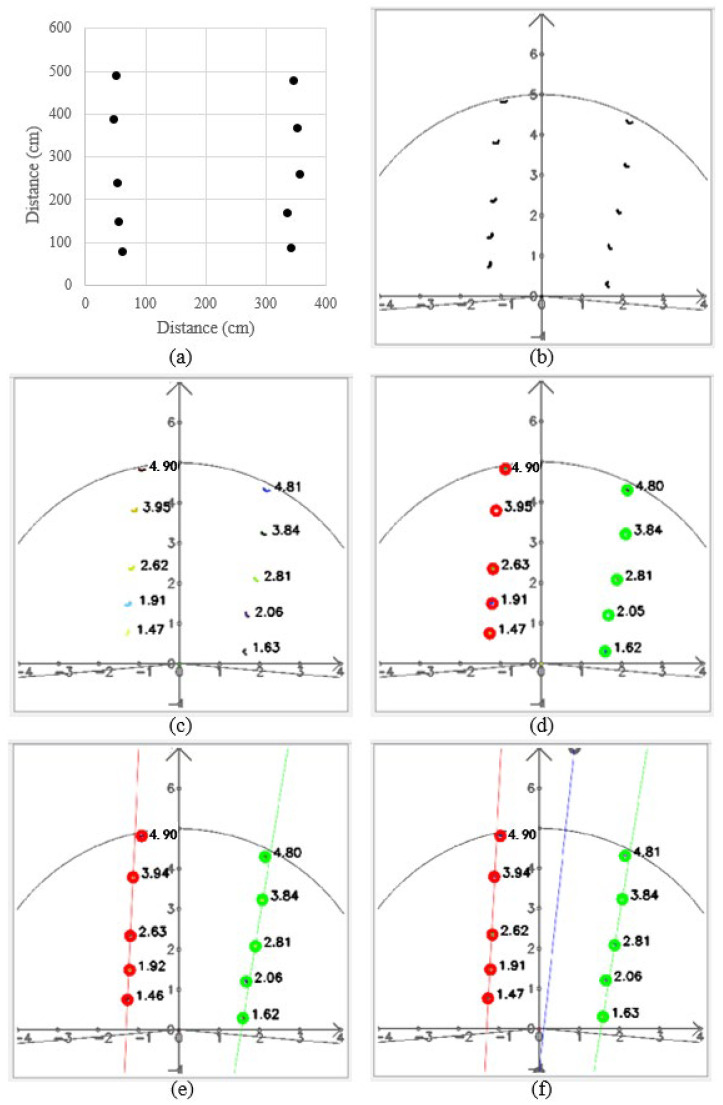
Path planning results: (**a**) cone positions; (**b**) data returned by LiDAR system; (**c**) DBSCAN results; (**d**) K-means results; (**e**) RANSAC results; and (**f**) the midline results.

In the calibration, the cones were placed randomly in two columns (Figure 12a). The vehicle was placed tilted, and the detection distance was set to 5 m. The points returned by the LiDAR system were only distance information, and the information was displayed in the interface through the calculation of the angle with the sending infrared signal (Figure 12b).

DBSCAN classified points into different clusters based on density; thus, it can accurately distinguish objects of different sizes (Figure 12c), and different clusters are shown as different colors. Therefore, this method can be used to detect different sizes of tree trunks in orchards. It was able to accurately cluster the points of each tree to distinguish the trunk locations.

The trees in the orchard are linearly arranged, and using K-means, the system can clearly classify the trees into left and right rows (Figure 12d); red is the tree on the left side, and green is the tree on the right side. While the vehicle was steered, the K-means algorithm could classify accurately. Because the scanning range was 5 m, even the clusters divided by DBSAN were not included in the path calculation.

After K-means classification, the points on the left and right sides were brought into the RANSAC algorithm to obtain the boundary lines on each side (Figure 12e). RANSAC could determine which cones were inline based on the location of the cones, and those cones could be used to calculate the path, while those outside the line would be regarded as noise points and not brought into the calculation; thus, this algorithm needed more than three points to obtain results. Finally, the midline was calculated as the planning path (Figure 12f).

### 3.2. Operation Calibration on a Concrete Road

#### 3.2.1. Calibration of the Curve Path

**Figure 13:**
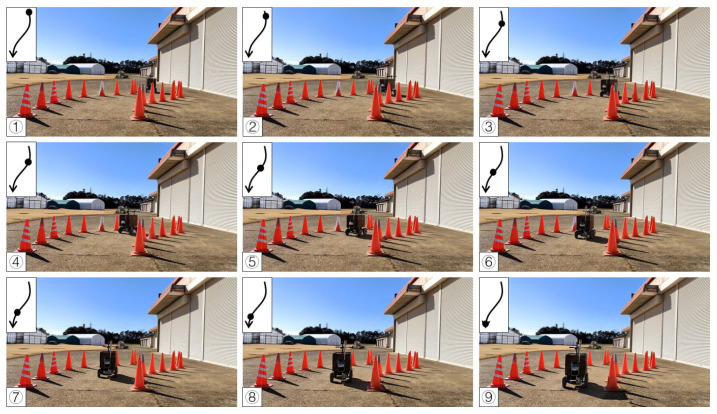
Curve path calibration on the concrete road (right turn). Subfigures (**1**–**9**) in order were the vehicle’s movement path.

**Figure 14:**
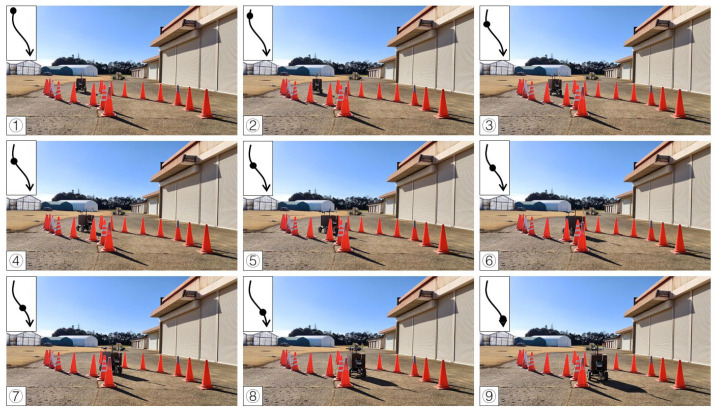
Curve path calibration on the concrete road (left turn). Subfigures (**1**–**9**) in order were the vehicle’s movement path.

To test the availability of the algorithm, the first calibration procedure was completed on a concrete road. A curved route was selected using 18 cones placed on two sides, and the vehicle was guided along the travel path based on the cone positions detected in real time. In the curve path correction, the detection distance of the LiDAR system was set to 2 m due to the close workshop. The radius ε of the circle of DBSCAN was set to 0.2 m and the minPoints was set to 3. The value of K for K-means was 2. The maximum operation times Max_Number of RANSAC was 300, the inline point proportion threshold (ProT) was 0.6, and the distance from the point to the line (DPL) was 0.05 m. The system calculated the steering angle by using the pure pursuit tracking method based on the objects’ position returned by the LiDAR system, iterated by incremental PID to obtain the current required steering angle and transmitted to Arduino. According to the experimental results, the vehicle reached the end point and turned in the right (Figure 13) and left directions (Figure 14).

**Figure 15:**
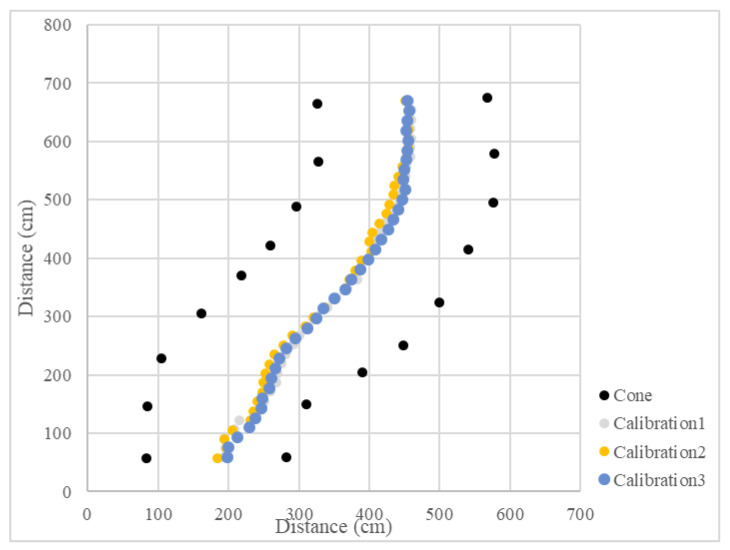
Operational path of the vehicle in the curve path calibration (right turn).

**Figure 16:**
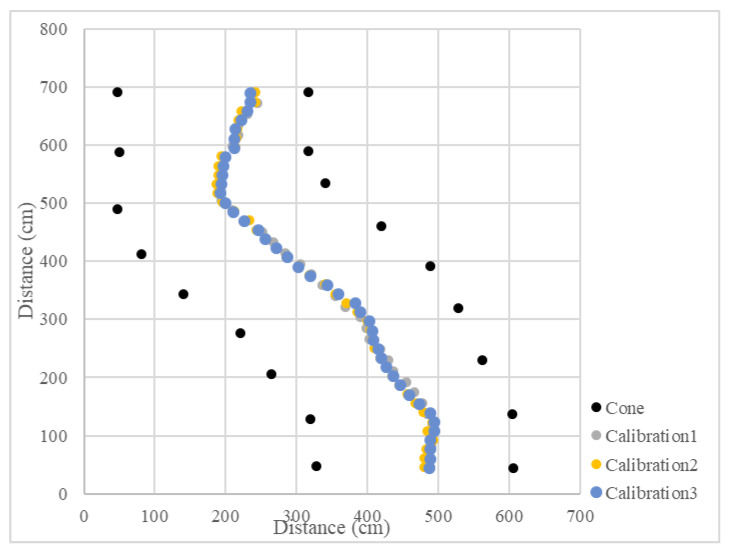
Operational path of the vehicle in the curve path calibration (left turn).

Since the LiDAR system was used as the single sensor, the program recorded the distance between the LiDAR system and the midline, as well as the steering angle and the operation radius during the U-turn and measured the position of the cones. The vehicle’s operational path was recovered (Figure 15 and Figure 16), which recorded the position of the cones and the results of three tests. The position root mean square errors (RMSEs) of the three right turn calibrations were 18.3 cm, 14.0 cm, and 18.9 cm, and the average value was 17.1 cm. The left turn calibration position RMSEs were 25.3 cm, 22.7 cm, and 24.3 cm, and the average value was 24.1 cm (Table 1). In orchard areas, navigation accuracy can be implemented for spraying operations.

#### 3.2.2. Calibration of Straight Maneuvers and U-Turns

**Figure 17:**
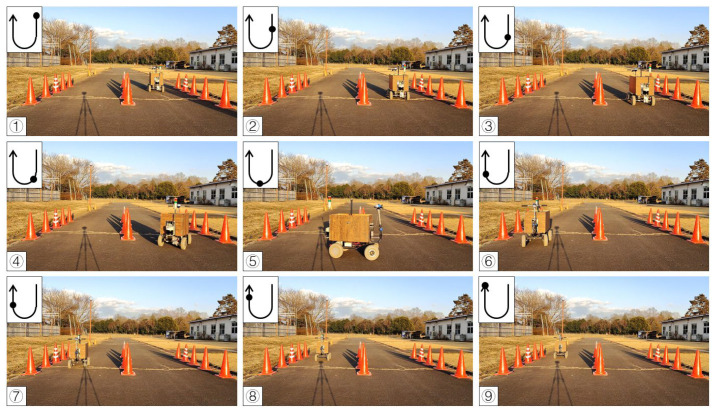
Images of vehicle operation on the concrete road (right turn). Subfigures (**1**–**9**) in order were the vehicle’s movement path.

**Figure 18:**
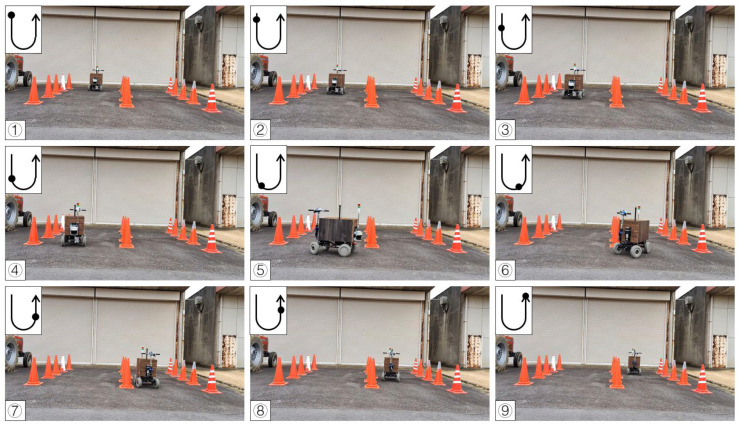
Images of vehicle operation on the concrete road (left turn). Subfigures (**1**–**9**) in order were the vehicle’s movement path.

**Figure 19:**
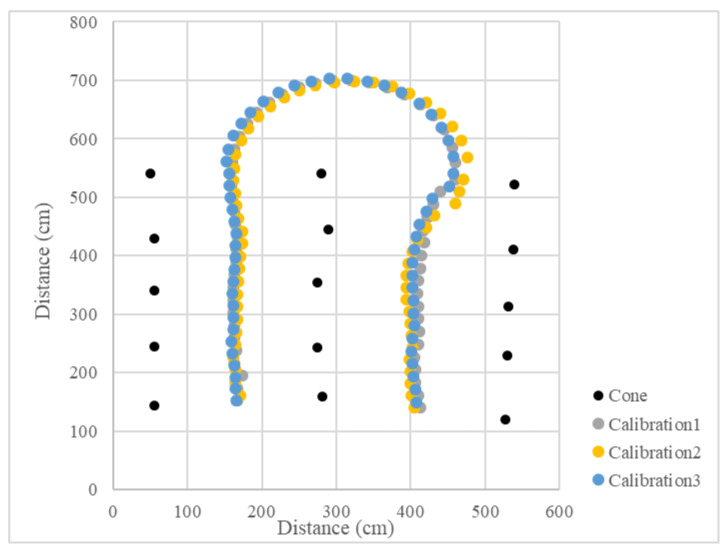
Operational path of the vehicle on the concrete road (right turn).

**Figure 20:**
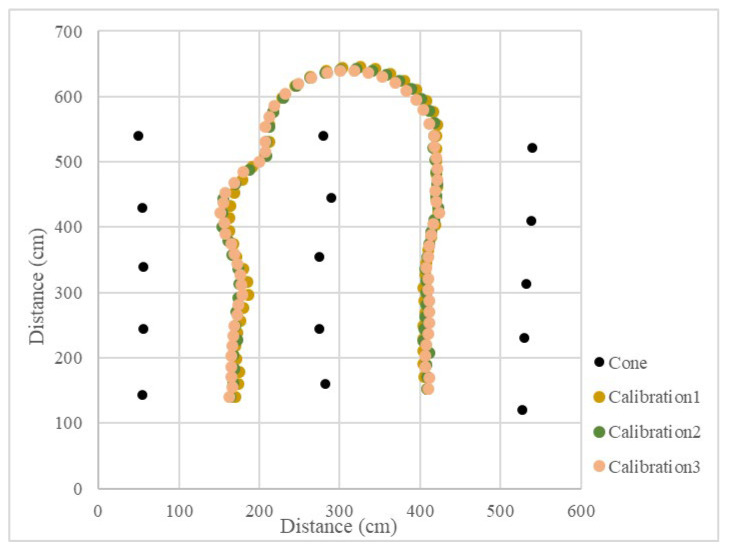
Operational path of the vehicle on the concrete road (left turn).

In the calibration test, 15 cones were placed in three columns to simulate the environment at the end of the orchard. In the test, the LiDAR detection distance was set to 3 m, only the cones on the left and right side could be detected, and the cones in the next column would not affect the planning path. Other parameters were kept the same. When the vehicle was moved in a straight line, the navigation system determined the planning path according to the real-time returned cone position data and moved along the path at a forward speed. When the number of cones on both sides was detected to be insufficient, the current position of the vehicle was close to the end of the orchard rows, and the vehicle would travel toward the midpoint of the last two cones. After reaching the midpoint, the steering angle was calculated based on the distance of the steering center cone, and the steering angle was maintained until reaching the second and third columns of the cones. When the LiDAR system detected enough scanning returned points again, the vehicle navigation system was enabled again using the LiDAR system. In the right-turn (Figure 17) and left-turn (Figure 18) tests, the cones were placed in the same position, and the vehicle was able to complete straight maneuvers and U-turns and stopped at the end of the path. The three routes were tested for right and left turns of the vehicle and recorded (Figure 19 and Figure 20). The three position RMSEs in the right turn calibrations on the concrete road were 10.4 cm, 14.6 cm, and 10.9 cm, and the average value was 12.0 cm. For the left turn, the calibrations were 12.0 cm, 11.2 cm, and 11.7 cm, and the average value was 11.6 cm (Table 1).

### 3.3. Operation Calibration on Grass

**Figure 21:**
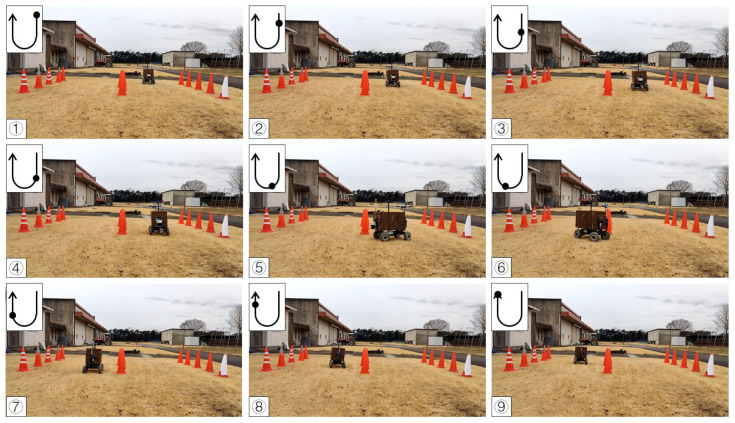
Images of vehicle operation on grass (right turn). Subfigures (**1**–**9**) in order were the vehicle’s movement path.

**Figure 22:**
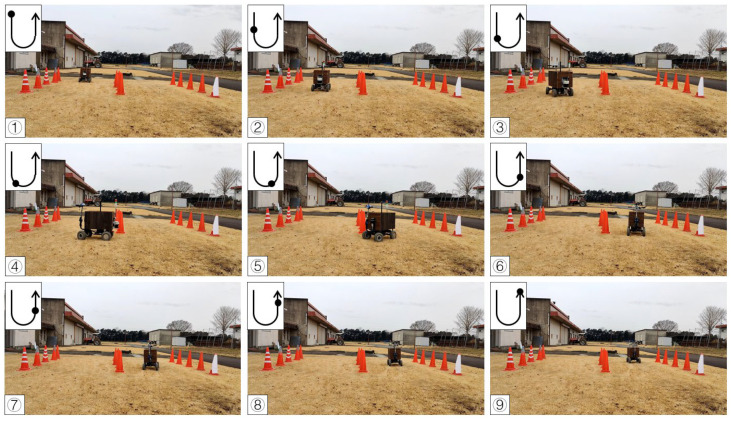
Images of vehicle operation on grass (left turn). Subfigures (**1**–**9**) in order were the vehicle’s movement path.

**Figure 23:**
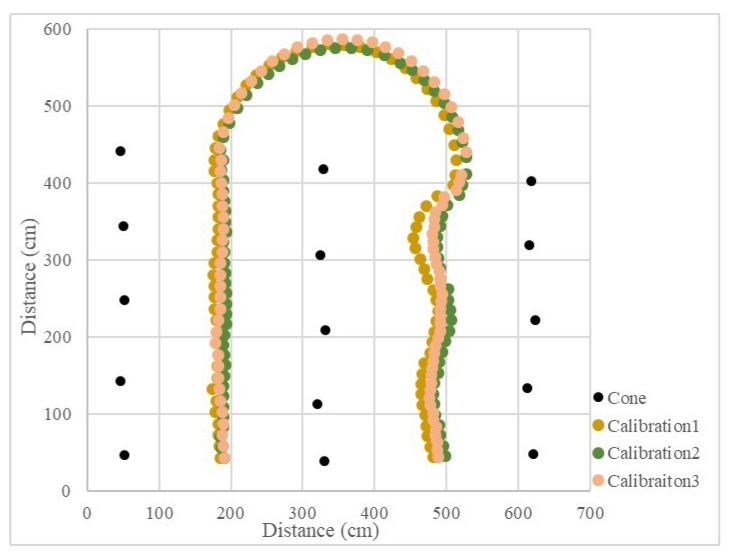
Operational path of the vehicle on grass (right turn).

**Figure 24:**
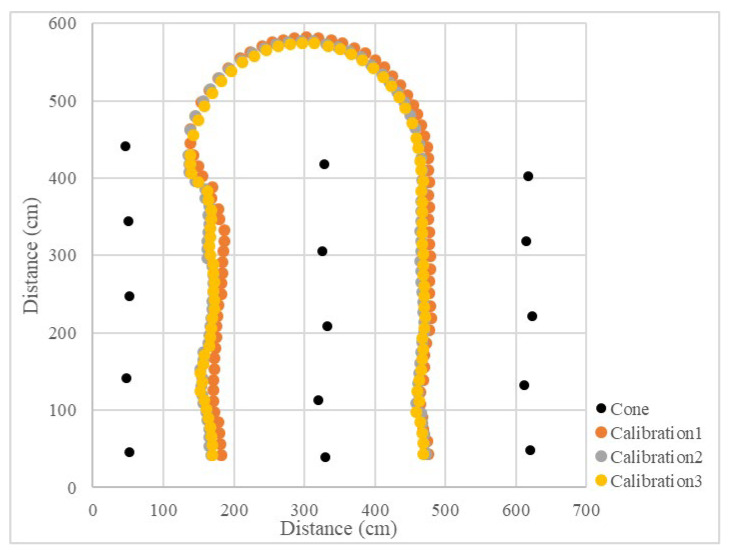
Operational path of the vehicle on grass (left turn).

To verify that the vehicle could operate properly in the orchard, calibration was also performed on grass before field testing. In the field, the motor speed was reduced while a driving test was conducted on the grass field to ensure that the motor had enough torque to steer. The experiment was the same as the concrete road, with three tests for each left turn, right turn (Figure 21 and Figure 22), and the recorded route (Figure 23 and Figure 24). The three position RMSEs in the right turn calibrations on grass were 9.4 cm, 15.8 cm, and 12.7 cm, and the average value was 12.6 cm. For the left turn, the calibrations were 10.7 cm, 18.6 cm, and 17.3 cm, and the average value was 15.5 cm (Table 1).

### 3.4. Field Test in a Facilitated Artificial-Tree-Based Orchard

**Figure 25:**
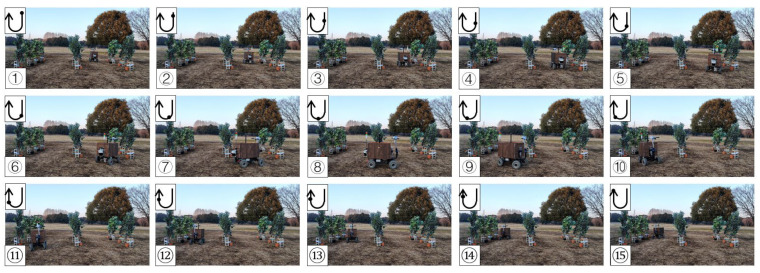
Images of vehicle operation in the facilitated artificial-tree-based orchard (right turn). Subfigures (**1**–**15**) in order were the vehicle’s movement path.

**Figure 26:**
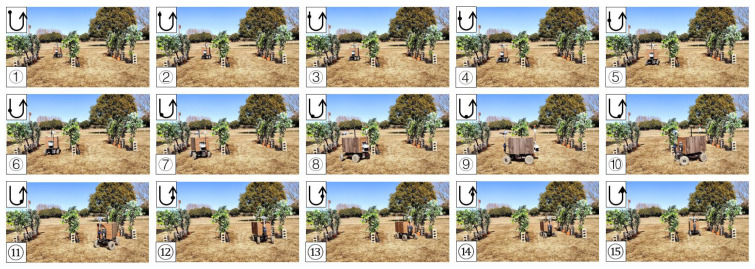
Images of vehicle operation in the facilitated artificial-tree-based orchard (left turn). Subfigures (**1**–**15**) in order were the vehicle’s movement path.

**Figure 27:**
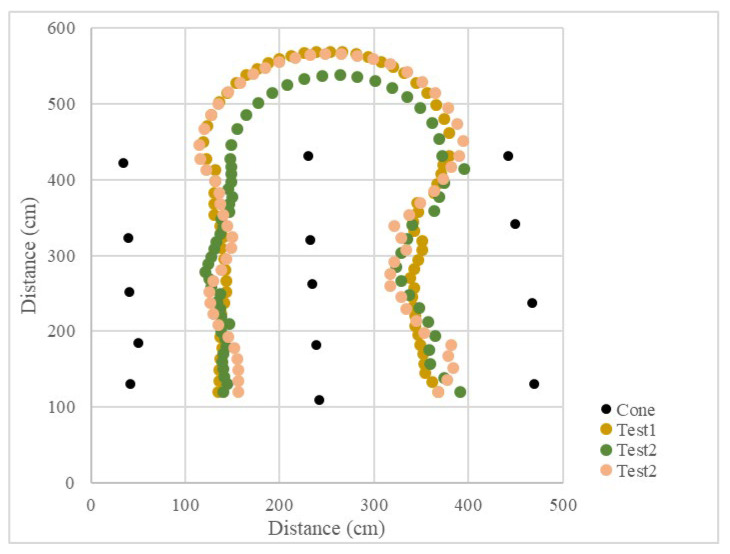
Operational path of the vehicle in the facilitated artificial-tree-based orchard (right turn).

**Figure 28:**
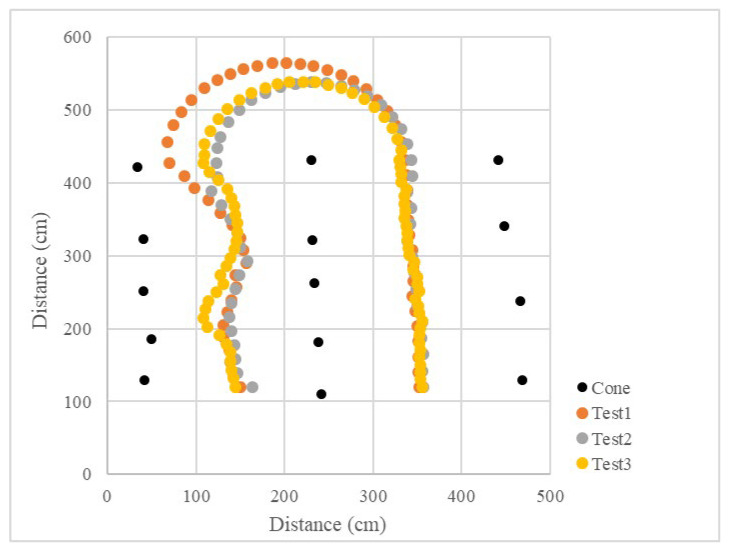
Operational path of the vehicle in the facilitated artificial-tree-based orchard (left turn).

The test was conducted in the Tsukuba Plant Innovation Research Center (T-PIRC), University of Tsukuba, facilitated in an orchard environment by placing artificial trees. The experimental site had a possible lack of trees, similar to natural orchards, and only four trees were used in the third column. However, the vehicle could still plan the correct route based on the algorithm (Figure 25, Figure 26, Figure 27 and Figure 28). The three position RMSEs in the right turn test in the facilitated artificial-tree-based orchard were 11.4 cm, 14.5 cm, and 15.5 cm, and the average value was 13.8 cm. For the left turn, the calibrations were 18.6 cm, 6.7 cm, and 8.8 cm, and the average value was 11.4 cm (Table 1).

## 4. Discussion

This study used LiDAR as a single sensor to achieve a stable navigation system in orchards compared to the RGB and GNSS systems. In the case of RGB images, vehicles are easily affected by light while driving in orchards and cannot accurately identify objects under low light conditions. The GNSS signals are interrupted due to the dense tree canopy in the orchard. LiDAR has advantages compared to RGB, and GNSS is not affected by light and signal quality in orchards. Furthermore, LiDAR has the potential to work at any time, providing a foundation for automatic navigation of the robot under different lighting conditions based on the DBSCAN, K-means, and RANSAC algorithms.

### 4.1. Machine Learning System from Point Clouds

Using the DBSCAN algorithm, the returned point clouds were divided into clusters based on density to identify the trunks. K-means divided the trunks into left and right groups and reduced the interference by RANSAC to obtain the appropriate boundary lines. The centerline was used as the output of the planning path result for the LiDAR-based navigation system.

### 4.2. Prototype Testing under the Different Lighting Conditions

**Figure 29:**
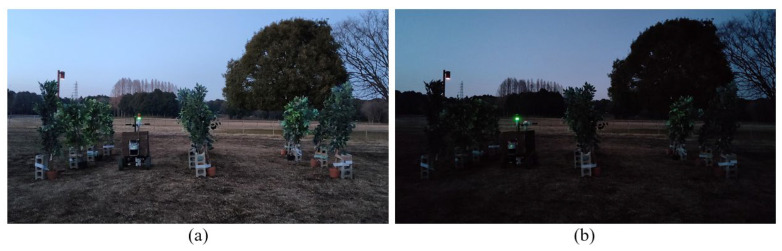
Experiment in the facilitated artificial-tree-based orchard under low-light conditions. (**a**) was experiment at 5–6 pm. (**b**) was experiment at 6–7 pm.

In the results section, the results are shown for sufficient light. To demonstrate that the navigation method proposed in this paper was not affected by the light conditions, the experiments were conducted under low-light conditions (5–7 pm) (Figure 29). Under low-light conditions, the LiDAR system could still make the vehicle move according to the position of the trees.

### 4.3. Prototype Testing on Concrete and Grass Using a Facilitated Artificial Tree Pattern

**Figure 30:**
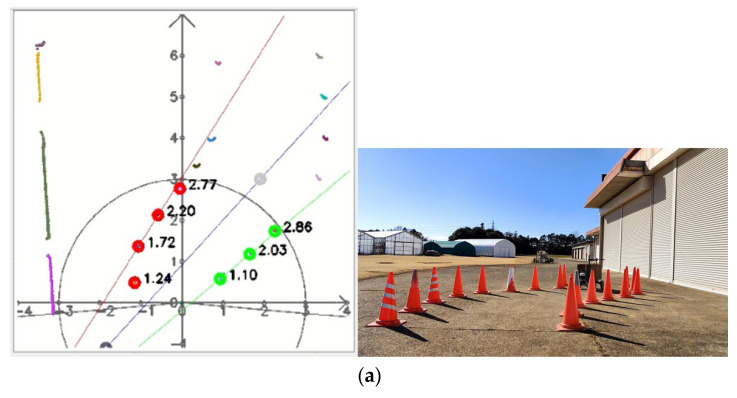

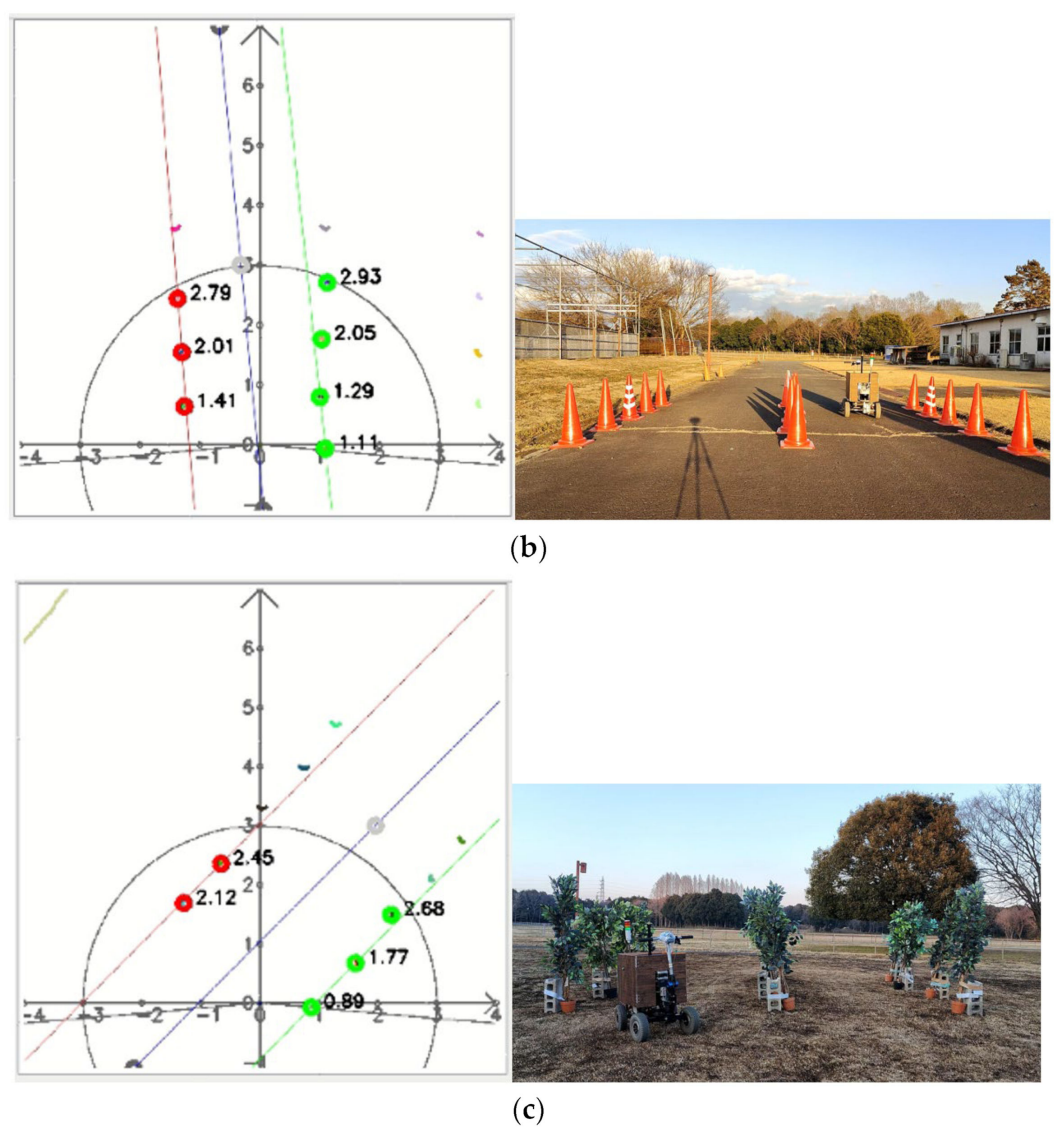
LiDAR visualization of the cluster datasets: (**a**) curve path on the concrete road, (**b**) straight maneuvers and U-turns on the concrete road, and (**c**) straight and U-turns on grass.

After testing on concrete, grass, and the facilitated artificial-tree-based orchard, the RMSE results were within 20 cm, indicating that the LiDAR system could operate safely and stably in those areas (Figure 30).

**Figure 31:**
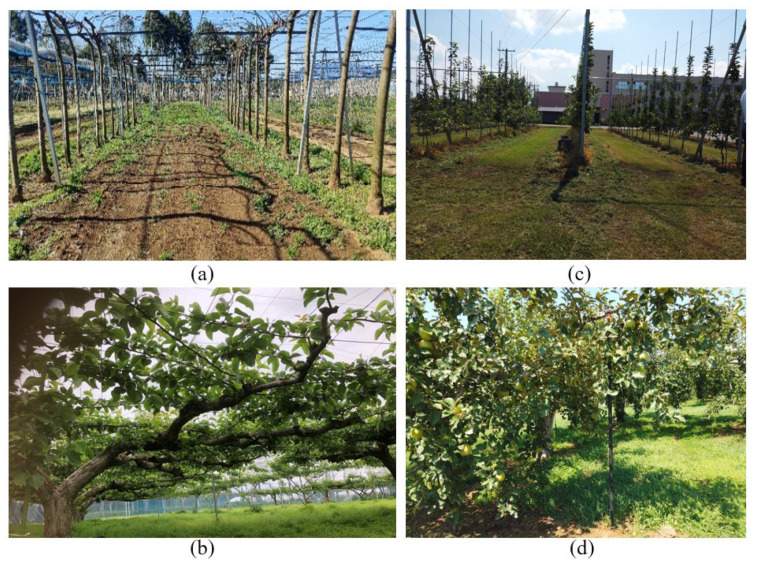
New trends and conventional orchards in Japan. (**a**) Initial stages of the joint tree training system for pears at the T-PIRC, (**b**) conventional orchard at the T-PIRC for pears covered with nets, (**c**) row-based vertical cordon training system at the apple research center in Aomori Prefecture in Japan, and (**d**) conventional apple orchard at the apple research center in Aomori Prefecture in Japan.

In the tests, while conducting turns on the concrete road, the three paths had minor differences compared to traveling on the designed path on grass and the facilitated artificial-tree-based orchard. This was due to the experimental vehicle using a stepper motor, which could affect the accuracy of the motor rotation on the ground where higher torque was needed. Therefore, during the experiment, the motor rotation speed was reduced, thus increasing the torque to reduce the error. However, even if there were errors during the U-turn, the vehicle could return to the planning path using PID control and reach the next row. In this research, the navigation method was suitable for orchards where trees were densely distributed. In recent times, Japanese pears have been grown in a systematic way, such as joint tree or row-based vertical cordon training practices, for ease of automation (Figure 31a,c).

In such joint tree systems, uniform shaped trees are grown in rows that can bring significant potential for the application of LiDAR to detect trees for path planning with higher accuracy. In our experiment, the tree-to-tree arrangement was placed at different distances within a range of 3 to 5 m. Since the trees were located 3 m apart and at least three tree locations were required to estimate the target position, LiDAR ranging was selected as 10 m from the vehicle. However, while choosing a 10 m scanning range, trees from adjacent rows were also added to the position estimation system, which affected the navigation accuracy.

### 4.4. Prototype Testing on Grass Using a Conventional Tree Pattern

**Figure 32:**
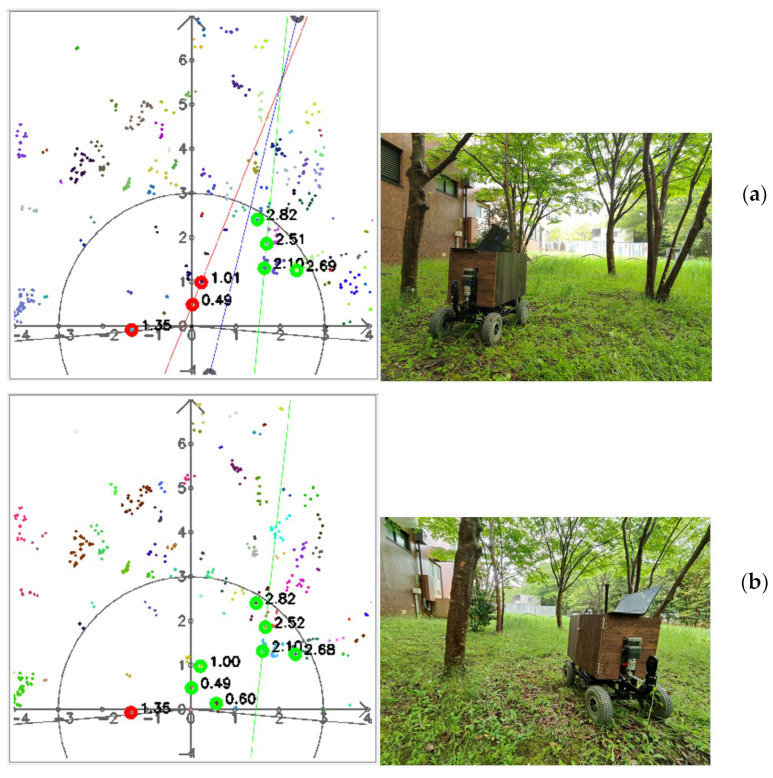
LiDAR visualization of cluster datasets in the actual environment: (**a**) correct path planning and (**b**) wrong path planning.

We tested the prototype and proposed LiDAR-based navigation system in the actual environment; however, due to sparse trees and the presence of high weeds, the navigation system was not consistent (Figure 32). With the development of agricultural automation, orchards have been changed to facilitate automated operations by planting trees as a column; however, since an actual orchard is being prepared, this study used artificial trees to facilitate the orchard environment in the T-PIRC experimental field.

If there were joint tree systems with a number of trees adjacent to each other, then the number of returned cloud points would be higher and high navigation performance could be achieved using the RANSAC algorithm. To facilitate such an environment, research was conducted to develop such a high-density orchard, and observing the navigation with only LiDAR is also possible in some limited conditions. However, to achieve more accurate navigation results, other visual sensors can be used in combination with LiDAR, such as thermal cameras, which also have high potential under low-light conditions for tree detection, as reported in our previous research [32]. To avoid weeds and external effects, deep learning is also reported with training and testing datasets of tree trunk for positioning reference. In further research, a combination of thermal-based vision sensors and LiDAR will be integrated to overcome the limitations of LiDAR for positional accuracy in the low-light conditions of orchards.

## 5. Conclusions

Orchard navigation is a challenging task due to signal interruptions by GNSS and illumination effects for contemporary vision systems. To overcome these limitations for orchard automation, LiDAR has the potential to be applied in low-light environments and GNSS signal interruption areas to obtain accurate distance information in real time. In this study, LiDAR was used as the only sensor to implement a navigation system in an orchard with the proposed point clouds data using machine learning principals. Therefore, the following points are outlined to conclude the contribution of this research and some of the limitations that will be addressed in future research:

1. Integration of machine learning algorithms, DBSCAN, K-means, and RANSAC, was performed to detect tree locations, divide them into left and right groups, and calculate boundary lines to find the midline for the navigation planning path.
2. Integration of the pure pursuit tracking algorithm and the incremental PID control were performed to calculate the steering angles for navigation. The calculated steering angles information was sent to the microcontroller to control the stepper motor rotation to achieve vehicle steering control on the navigation path in real-time.
3. In the concrete road calibration, the positional RMSE in the curve and the U-turn calibrations were 20.6 cm and 11.8 cm, respectively, indicating that the guidance system could calculate the path and control the vehicle safely based on the position of the landmarks in real time.
4. In the grass calibration, the position RMSE of the right and left turns was 14.1 cm, proving that this navigation system could operate properly on the soil. In the facilitated artificial-tree-based orchard, the positional RMSE was 12.6 cm, and a U-turn was performed to steer the robot when applying pesticides in the joint-orchard system for our future research.
5. In this study, an automatic navigation system for orchards was produced using only 2D LiDAR. Not only can the vehicle be driven under any light conditions, but the computational complexity was also reduced; thus, it does not need to rely on powerful performance computers.

Therefore, LiDAR can be used in limited conditions as a single sensor for navigation inside orchards. Since LiDAR can only determine the distance of the object and cannot distinguish the type of object, it has certain requirements to facilitate the environment. Compared with conventional orchard, the accuracy of joint orchard with closely planted fruit trees are get higher accuracy. However, for flexibility in application in different environments in orchards, thermal-vision-based sensors, which are robust under low-light conditions, can also be integrated. Therefore, in future research, LiDAR and thermal cameras can be integrated to provide solutions for orchard robot spraying systems from different infrastructure perspectives to increase the productivity of orchard operational management.

## Figures and Tables

**Table 1:** RMSE results for each calibration and field experiment.

Site		RMSE (cm)	Calibration 1	Calibration 2	Calibration 3	Average
	Path					
Concrete road	Curve path (right)		18.3	14.0	18.9	17.1
	Curve path (left)		25.3	22.7	24.3	24.1
	Operational path (right)		10.4	14.6	10.9	12.0
	Operational path (left)		12.0	11.2	11.7	11.6
Grass	Operational path (right)		9.4	15.8	12.7	12.6
	Operational path (left)		10.7	18.6	17.3	15.5
Facilitated artificial-tree-based orchard	Operational path (right)		11.4	14.5	15.5	13.8
	Operational path (left)		18.6	6.7	8.8	11.4

## Data Availability

The datasets generated and analyzed during this study are available from the corresponding author upon reasonable request, but restrictions apply to the data reproducibility and commercially confident details.
